# The *Chlamydiales* Pangenome Revisited: Structural Stability and Functional Coherence

**DOI:** 10.3390/genes3020291

**Published:** 2012-05-16

**Authors:** Fotis E. Psomopoulos, Victoria I. Siarkou, Nikolas Papanikolaou, Ioannis Iliopoulos, Athanasios S. Tsaftaris, Vasilis J. Promponas, Christos A. Ouzounis

**Affiliations:** 1 Institute of Agrobiotechnology, Centre for Research & Technology Hellas (CERTH), Thessaloniki GR-57001, Greece; E-Mails: fpsom@certh.gr (F.E.P.); tsaft@certh.gr (A.S.T.); 2 Department of Electrical & Computer Engineering, Aristotle University of Thessaloniki, Thessaloniki GR-54124, Greece; 3 Laboratory of Microbiology & Infectious Diseases, Faculty of Veterinary Medicine, Aristotle University of Thessaloniki, Thessaloniki GR-54124, Greece; E-Mail: vickysi@vet.auth.gr; 4 Division of Medical Sciences, University of Crete Medical School, Heraklion GR-71110, Greece; E-Mails: papnikol@med.uoc.gr (N.P.); iliopj@med.uoc.gr (I.I.); 5 Department of Genetics & Plant Breeding, Aristotle University of Thessaloniki, Thessaloniki GR-54124, Greece; 6 Bioinformatics Research Laboratory, Department of Biological Sciences, University of Cyprus, P.O. Box 20537, Nicosia CY-1678, Cyprus; E-Mail: vprobon@ucy.ac.cy; 7 Donnelly Centre for Cellular & Biomolecular Research, University of Toronto, 160 College Street, Toronto, Ontario M5S 3E1, Canada

**Keywords:** comparative genomics, pangenome analysis, *Chlamydiales*, protein family detection, genome annotation, genome trees

## Abstract

The entire publicly available set of 37 genome sequences from the bacterial order *Chlamydiales* has been subjected to comparative analysis in order to reveal the salient features of this pangenome and its evolutionary history. Over 2,000 protein families are detected across multiple species, with a distribution consistent to other studied pangenomes. Of these, there are 180 protein families with multiple members, 312 families with exactly 37 members corresponding to core genes, 428 families with peripheral genes with varying taxonomic distribution and finally 1,125 smaller families. The fact that, even for smaller genomes of *Chlamydiales*, core genes represent over a quarter of the average protein complement, signifies a certain degree of structural stability, given the wide range of phylogenetic relationships within the group. In addition, the propagation of a corpus of manually curated annotations within the discovered core families reveals key functional properties, reflecting a coherent repertoire of cellular capabilities for *Chlamydiales*. We further investigate over 2,000 genes without homologs in the pangenome and discover two new protein sequence domains. Our results, supported by the genome-based phylogeny for this group, are fully consistent with previous analyses and current knowledge, and point to future research directions towards a better understanding of the structural and functional properties of *Chlamydiales*.

## 1. Introduction

Members of the order *Chlamydiales* are obligate intracellular bacteria, characterized by a unique developmental cycle and are important pathogens of humans and animals resulting in a wide range of diseases, including several zoonoses [1,2,3]. The order *Chlamydiales*, separated from other eubacteria by forming a deep branch in ribosomal RNA-based phylogenetic trees, has been enriched by new lineages. Beside the family *Chlamydiaceae*, in which important chlamydial pathogens are grouped, new families, such as *Parachlamydiaceae*, *Simkaniaceae* and *Waddliaceae*, have been recognized to accommodate newly discovered pathogenic and non-pathogenic chlamydial organisms [4,5,6].

Since the release of the first chlamydial genome sequence from *Chlamydia trachomatis* (serovar D) [7], new genomes are being sequenced, thus offering insights into the genome organization and functional capacity of the corresponding species [8]. Besides its crucial importance for applied research in medical and veterinary microbiology [9], this corpus of genomic information is also key to understanding the evolutionary position of various chlamydial species (or strains) and the inference of the internal phylogeny of this distinct taxon [8,10,11,12].

As the intracellular lifestyle imposes constraints on gene content and metabolic capabilities, the *Chlamydiales* might represent one of the best datasets for the development of pangenome analysis methods [13]. Additional challenges are the wide variety of chlamydial genome sizes with unequal rates of reduction, and a repertoire of less characterized proteins than other bacterial groups whose pangenomes have been analyzed, e.g., *Streptococcus* or *Salmonella* [14,15].

Previously, we have used the genome of *Chlamydia trachomatis* [7] as a case study for annotation transfer quality [16]. Using a novel encoding scheme and a scoring function called TABS for transitive annotation-based scale [16], our main finding regarding annotation was that, despite a number of inconsistencies, automated annotation pipelines performed remarkably well when benchmarked against a manually curated annotation corpus [16]. These results are important for the quantification of reproducibility and consistency in genome-wide annotation [17].

In this work, we explore the entire set of the *Chlamydiales* pangenome with a broad collection of genome sequences publicly available to date (31 *Chlamydiaceae* and six other *Chlamydiales* genomes), twice as many as in a similar recent analysis [18]. Importantly, our pangenome analysis pipeline incorporates recently sequenced genomes of key *Chlamydiaceae* species not previously reported, thus augmenting our understanding from previous findings [18,19].

We focus on key aspects of pangenome analysis and explore multiple facets of the *Chlamydiales* gene content in terms of protein-coding genes and families. We also provide certain key findings that might illuminate the evolutionary history of this group as well as interesting sequence motifs not widely shared within this order. Beyond the confirmation of the recent analysis of the *Chlamydiales* as mentioned above [18], we also use this group to expand on methods for pangenome analysis [13,20] by proposing a pangenome analysis pipeline. Our results are consistent with wider studies of pangenomes [21] and provide additional knowledge for *Chlamydiales*. In conclusion, pangenome analysis offers an opportunity for the study of bacterial genome evolution, the development of relevant methods and the understanding of genome structure and proteome function on a large scale.

## 2. Experimental Section

### 2.1. Data Collection

All protein sequence data from 37 genomes were compiled into a single data collection (February–July 2011), including the most recent published *Chlamydiales* genomes. In total, 43,736 protein-coding genes were extracted from public databases corresponding to the entire set of 37 genome sequences from the bacterial order *Chlamydiales* currently available (Table 1). Sequence data were codified following the style of the COGENT database [22], for easy identification both by programs and human users (Supplement S1). The above notation is followed throughout this work. The COGENT scheme encodes genus and species names into a four-character identifier prefix string, followed by a code for the strain name, its version (in this collection all versions are considered as version 1 and optionally hidden) and finally for proteins the relative order of the sequence within the genome [23] (Table 1). We have also recorded the date of publication for the corresponding genome (or the release date where no publication was available) (Supplement S2).

**Table 1:** List of *Chlamydiales* genome sequences used in this study.

##	Species and Strain Name/Codes	Internal Identifier	Protein-Coding Genes
01	*Candidatus Protochlamydia amoebophila* UWE25	CPRO-UWE-01	2,031
02	*Chlamydia muridarum* Nigg	CMUR-NIG-01	911
03	*Chlamydia trachomatis* 434/Bu	CTRA-434-01	874
04	*Chlamydia trachomatis* A/HAR-13	CTRA-AHA-01	919
05	*Chlamydia trachomatis* B/Jali20/OT	CTRA-BJA-01	875
06	*Chlamydia trachomatis* B/TZ1A828/OT	CTRA-BTZ-01	880
07	*Chlamydia trachomatis* D/UW-3/CX	CTRA-DUW-01	895
08	*Chlamydia trachomatis* L2b/UCH-1/proctitis	CTRA-L2B-01	874
09	*Chlamydophila abortus* S26/3	CABO-S26-01	932
10	*Chlamydophila caviae* GPIC	CCAV-GPI-01	1,005
11	*Chlamydophila felis* Fe/C-56	CFEL-FEC-01	1,013
12	*Chlamydophila pneumoniae* AR39	CPNE-AR3-01	1,112
13	*Chlamydophila pneumoniae* CWL029	CPNE-CWL-01	1,052
14	*Chlamydophila pneumoniae* J138	CPNE-J13-01	1,069
15	*Chlamydophila pneumoniae* TW-183	CPNE-TW1-01	1,113
16	*Waddlia chondrophila* WSU 86-1044	WCHO-WSU-01	1,956
17	*Chlamydia trachomatis* E/150	CTRA-E15-01	927
18	*Chlamydophila pecorum* E58	CPEC-E58-01	988
19	*Chlamydophila psittaci* 6BC	CPSI-6BC-01	975
20	*Chlamydophila abortus* LLG	CABO-LLG-01	925
21	*Chlamydophila pneumoniae* LPCoLN	CPNE-LPC-01	1,105
22	*Chlamydophila psittaci* Cal10	CPSI-CAL-01	1,005
23	*Parachlamydia acanthamoebae* UV7	PACA-UV7-01	2,788
24	*Parachlamydia acanthamoebae* str. Hall’s coccus	PACA-HAL-01	2,809
25	*Simkania negevensis* Z	SNEG-ZXX-01	2,518
26	*Waddlia chondrophila* 2032/99	WCHO-203-01	2,015
27	*Chlamydophila psittaci* 01DC11	CPSI-01D-01	975
28	*Chlamydophila psittaci* 02DC15	CPSI-02D-01	978
29	*Chlamydophila psittaci* 08DC60	CPSI-08D-01	973
30	*Chlamydia trachomatis* D-EC	CTRA-DEC-01	878
31	*Chlamydia trachomatis* D-LC	CTRA-DLC-01	878
32	*Chlamydia trachomatis* E/11023	CTRA-E11-01	926
33	*Chlamydia trachomatis* G/11074	CTRA-G74-01	919
34	*Chlamydia trachomatis* G/11222	CTRA-G22-01	927
35	*Chlamydia trachomatis* G/9301	CTRA-G93-01	921
36	*Chlamydia trachomatis* G/9768	CTRA-G97-01	920
37	*Chlamydia trachomatis* Sweden2	CTRA-SWE-01	875
		Total	43,736

The first column signifies the inclusion order into the genome collection and does not reflect any other relationship. The second column lists the species and strain name, the third column the COGENT-style identifier and the last column the number of protein-coding genes.

### 2.2. Sequence Comparison

All protein sequence data were masked using CAST with default parameters (threshold = 40), to exclude compositionally biased regions [24]. In total, 6,906 such regions were filtered out, provided for further study (Supplement S3).

The masked sequences were used as queries against the genome corpus, in an all-against-all mode with BLAST (blastall, e-value threshold 10^−6^) [25,26]; in total, more than 40,000 BLAST searches were performed and 1,709,325 significant similarities below threshold were obtained (Supplement S4).

### 2.3. Clustering and Annotation

The similarity pairwise list (from Supplement S4) was submitted to MCL sequence clustering [27], with default parameters (e.g., inflation value 2.0); clusters were incrementally assigned to an integer identifier. Clusters are sorted by their size (number of members in a cluster, Supplement S5); thus, the largest clusters have smallest-integer identifiers (see Results and Appendix-Table 1). This approach has also been used successfully elsewhere [28] as a method of choice.

Annotation transfer based on the first chlamydial genome ever sequenced was implemented through the direct matching of the lead sequences to a previously highly curated dataset for *Chlamydia trachomatis* D/UW-3/CX [7].

The annotation qualifiers used in the manually curated corpus [16] are: ENZYME (for enzymes with EC number assignments), FUNCTION (for other protein functions), SIMILAR-TO (for those sequences with a similarity to a protein of known function but no specific assignment) and DOMAIN (for the existence of a known, named protein sequence domain) [16] (Appendix-Table 1).

Sequence matching of the original dataset to the data collection presented here was performed by MagicMatch [29], which was the first scheme to implement the MD5 checksum for protein sequence identification, an approach later propagated in all major database resources.

### 2.4. Analysis of Unique Genes

All unique genes, *i.e*., more than 2,000 genes with no similarity within the pangenome, were searched against the non-redundant protein sequence database (nrdb: 15,052,178 entries) [30]. Results from this search were evaluated manually and key similarities were extracted for further investigation (Supplement S6).

### 2.5. Genome Trees

Genome-based trees were calculated using phylogenetic profile distance [31,32]. Similarity values were measured by the shared number of genes represented by phylogenetic profiles, symmetrified by the minimum shared value, normalized by minimum self-similarity and turned into distance values as previously described [32,33] (Supplement S7).

### 2.6. Sequence Alignments

Multiple sequence alignments were performed and visualized by JalView [34]. Novel motifs reported in this work are provided below and in FASTA format (Supplements S8,9).

### 2.7. Data Availability

Per genome contributions to the pangenome are also provided (Supplement S10). All sequence data and results (in 10 Supplements) have been made available at datadryad.org, under the identifier [35].

## 3. Results

### 3.1. General Characteristics of the Chlamydiales Pangenome

The *Chlamydiales* collection herein contains over 40,000 protein-coding genes in total, with ~1,200 genes/genome on average, with significant deviations (Table 1). We take the view to present the two extreme tails of this data collection in detail following the clustering step for the identification of protein families within the pangenome and comment on the intermediate cases. In other words, we primarily focus on the two classes of the most interesting clusters, (i) those containing the core genes and (ii) those corresponding to “unique” genes, without significant similarities within the pangenome, thus singleton clusters. The functional characterization of the entire complement as well as further issues listed in the discussion for future research are clearly beyond the scope of this critical review.

### 3.2. Protein Families

**Figure 1:**
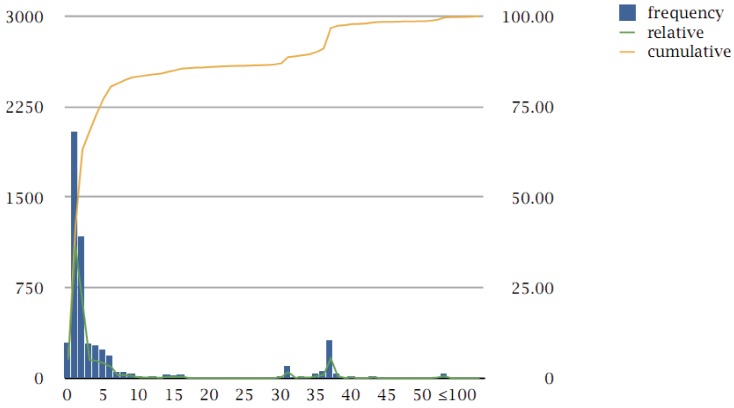
Pangenome protein family size distribution. Cluster size is displayed on the x-axis (bins until 50 are all shown; above 50, bins are shown for each ten counts, labels for every five bin sizes); absolute frequency of clusters is shown on the left y-axis (bars, green curve); cumulative count of clusters is shown on the right y-axis (orange curve). Families are defined as those clusters with at least three members (see text); all cluster frequencies are shown here for completeness. The bimodal nature of the distribution can be seen between the peak at low cluster sizes and 37; above 37 there are multi-member and multi-species protein families (see text).

In total, the clustering has yielded 5,554 clusters corresponding to protein families. For practical purposes, we define a protein family as one that contains at least three genes: in that sense, there are 294 cases, which do not detect themselves in this comparison (typically because of either short length, abnormal composition, or both), 2,038 unique genes (singletons) and 1,177 doublets. The remaining 2,045 clusters represent protein families with three or more members, distributed across 37 genomes (Figure 1).

It is evident that the protein family size distribution follows, as expected, the shape of other pangenome analyses, with a clear bimodal distribution, with one peak at low-count families which has been called the “accessory pool” and another peak at the limit of the genomes under consideration, which has been called the “extended core” [21]. The so-called “character genes” (which we prefer to define as “peripheral”, as opposed to “core” genes) exhibit, by definition, a heterogeneous distribution across genomes (and between peaks) and present an additional challenge for further interpretation (Figure 1).

The peak at exactly 37 with 312 counts, *i.e*., 312 families with exactly 37 members, corresponds to the number of 37 genomes analyzed across the pangenome. Beyond that peak, there are 180 protein families with more than 37 members (clusters 1–180) (Supplement S5), of which ten contain more than 100 members and are discussed below.

### 3.3. Multi-Member Families

**Figure 2:**
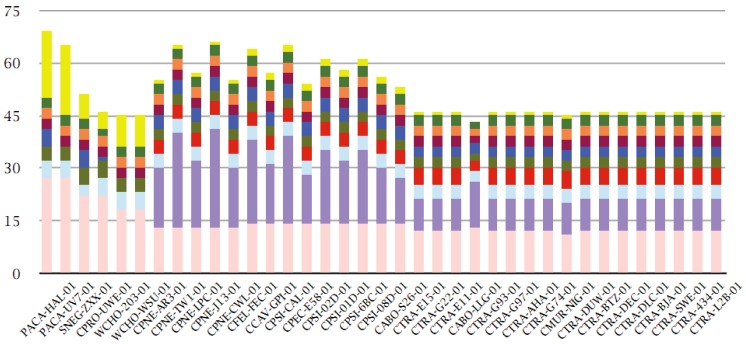
Top ten multi-member families within the pangenome. Genomes (with full COGENT-like codes) are shown on the x-axis, sorted by total protein-coding gene count (see also Table 1). Absolute cumulative counts of multi-member families are shown on the y-axis (displayed in the figure legend from left to right and then top to bottom, e.g., ABC transporter permeases, POMPs, type III secretion system ATPases, etc. according to size, see text), color coded according to figure legend.

The four largest families with more than 120 members are represented by the ABC transporter permeases (530 members), the polymorphic outer membrane proteins of *Chlamydiaceae* [36] (POMPs, 435 members), the flagellum-specific ATP synthases/type III secretion system ATPases, e.g., CT669 [37] (152 members), and a family of unknown function recently characterized as type III secreted effectors [38] (DUF582, 140 members) (Figure 2).

Following those, there are another four families with more than 110 members each: the EF-Tu/EF-G/LepA family (119 members), the oligopeptide binding protein family OppA (114 members), the GroEL family (111 members) and finally the Ile-Leu-Val (ILV)-tRNA synthetases (111 members). These are followed by two families with more than 100 members, namely the Dihydrolipoamide acetyltransferase E2 component/Dihydrolipoamide succinyltransferase (110 members) and the 3-oxoacyl-[acyl-carrier protein] reductase families (109 members) (Figure 2).

A significant number of multi-member families contain proteins of known function (Supplement S5). Interestingly, families containing only homologues from *S. negevensis*, *W. chondrophila*, *P. acanthamoebae* and *Protochlamydia amoebophila* are 172 in total, remarkably close to the 171 clusters of “orthologous” proteins in this group of species reported recently [18].

### 3.4. Core Genes

At the other end of the bimodal distribution, there are 312 families with 37 genes each, reflecting the number of genomes analyzed. However, there are eight clusters here with duplicates per genome (clusters 224, 460: *S. negevensis*; 254, 276, 420: *P. acanthamoebae*; 255, 272: *P. amoebophila*; 429: *W. chondrophila* 203) (two of which of unknown functional roles, Appendix-Table 1). Thus, there are exactly 304 protein families with 37 genes each represented once in each genome, which can be truly called “core” genes, most of which have some source of annotation (Appendix-Table 1). These represent just over a quarter of the average chlamydial genome (304/1182 = 26%).

Annotations transferred from the manually curated seed annotation corpus of *C. trachomatis* reveal a wide range of functional roles for this core set, as expected (Appendix-Table 1). Indeed, 227 families of the core set can be assigned to a functional role, according to the annotation qualifiers originally used (see Experimental Section). Only an additional 77 cases in this set do not contain any annotation (Appendix-Table 1). It can be argued, therefore, that this level of characterization of 75% (227/304) across 37 genomes signifies a functional coherence that is consistent with our current knowledge of this taxonomic order. This list is provided for further investigation by the community; it is worth pointing out that it encompasses basic cellular roles in genetic information processing (e.g., cluster 184), including transcription (e.g., cluster 187) and translation (e.g., clusters 242–243), metabolic transformations (e.g., cluster 182 or 196), transport systems (e.g., clusters 193–195) and other key processes (e.g., cluster 192). It is interesting to note that apart from complements represented by ribosomal proteins or aminoacyl-tRNA synthetases, other systems are also coherently detected, for example the NifU [39]/NifS [40] genes (clusters 221–222).

### 3.5. Peripheral Genes

In the midst of the two extremes (viz. peaks) of the bimodal family size distribution, there exists a wide variety of cases with an anomalous and clearly heterogeneous pattern. There are 428 families with more than ten and less than 37 members (not shown, available in Supplement S5). Their hererogeneous composition is reflected by the fact that 217 of the 428 families (just over 50%) do not contain a homolog outside the *Chlamydiaceae*, *i.e*., across the larger genomes mentioned above. Within this group, however, there is a significant variation of family phylogenetic distribution (not shown) that needs to be explored in future research.

### 3.6. Unique Genes

**Figure 3:**
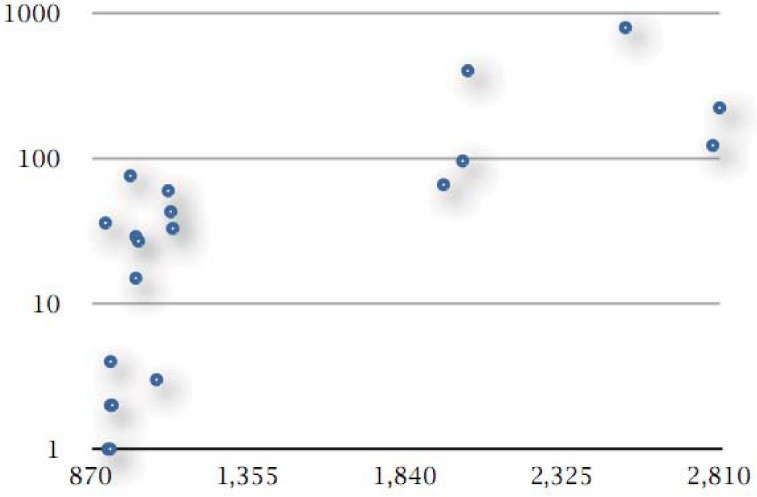
Correlation between genome size and unique genes. Genome size is given as the number of protein-coding genes (shown on the x-axis) against the count of unique genes (number of unique genes without homologs within the pangenome, shown on the y-axis; y-axis is displayed on logarithmic scale). The six points on the upper right part of the graph are evidently those genomes with largest gene counts, all outside the *Chlamydiaceae* family (see Table 1 and text). The pattern observed is primarily due to the sampling of taxonomic space of the *Chlamydiales* and will vary as more genomes from this group become available.

In total, there are 2,038 unique genes represented by singleton clusters, thus not falling into families within the pangenome. The content of genomes with unique genes varies significantly, from 0 to 796 (*S. negevensis*), with 55 unique genes on average. In percentage points, this varies from obviously 0 to 32% of the genome (*S. negevensis*), with an average of just over 3% per genome (Figure 3).

The densest part of the phylogeny exhibits no unique genes—17 genomes, including most of the *C. trachomatis* and *C. psittaci* strains, *C. pneumoniae* CWL029 and *C. abortus* LLG (Figure 3, missing points corresponding to 17 genomes with zero value on the y-coordinate, available in Supplement S5). Twenty genomes have unique genes, of which six genomes have less than 10 such genes and one with 15 unique genes (Figure 3), all from the above group, or less than 2% of their genome entries. Another five genomes with a handful of unique genes are *C. pneumoniae* AR39 (33/3%), TW-183 (43/4%) and LPCoLN (60/5%) as well as *C. felis* (27/3%) and *C. caviae* (29/3%). The remaining eight genomes contain the majority of unique genes, 1818 in number or 89% of total, ranging from 66 (*W. chondrophila* WSU, 3% of genome) to 796 genes (*S. negevensis*, 32% of genome). This is not entirely a biological effect, rather a sampling artifact arising from the deeper sequencing of the *C. trachomatis/C. pneumoniae* group (see below).

The six outliers which form a different group above (upper right, Figure 3) are all species with large genomes (*ca*. 2,000 protein-coding genes or more): the two *W. chondrophila* strains (3–4%), the two *P. acanthamoebae* strains (4–8%), *P. amoebophila* (20%), and *S. negevensis* (32%), listed here according to the absolute number of their unique genes per genome. In relative terms, however, two species namely *C. muridarum* (36/4%), and *C. pecorum* (76/8%) contain a significant number of unique genes given their relatively small genome size (both less than 1,000 protein-coding genes).

### 3.7. Properties of Unique Genes

The genes considered as singletons in this analysis are 2,038 as mentioned above. Of those, a number of short genes might fall into pangenome families (not shown) but do not seriously affect the overall assessment (e.g., case CCAV-GPI-01-000824 in Supplement S6). This is an artifact of sensitivity for the two different searches, first against the 40,000 or so genes of the pangenome and second against the entire nrdb database of more than 15 million sequences. While a full analysis of the unique gene complement of the *Chlamydiales* is under progress, it is interesting to report on a number of findings pertinent to this work.

A number of genes from the pangenome have identified homologs such as cell-wall associated hydrolases (TC0114 from *C. muridarum* Nigg), proteins of unknown function (e.g., pc0061, pc0549, pc0850, pc0855), endonucleases (e.g., pc0252), exonucleases (pc0951), transposases (e.g., pc0068), DNA repair proteins (e.g., pc0286), acyltransferases (e.g., pc0180), Mg chelatases (pc0480), oxidoreductases (pc0504), streptomycin 6-kinases (e.g., pc0510), metallophosphoesterases (pc0948) from *P. amoebophila* and LmbE/ypjG family proteins (e.g., wcw_0275) or transposases (e.g., wcw_0482) from *W. chondrophila* WSU. Similarly, multiple cases of similarity to families of known or unknown function are discovered for unique genes from the larger genomes (not shown).

**Figure 4:**
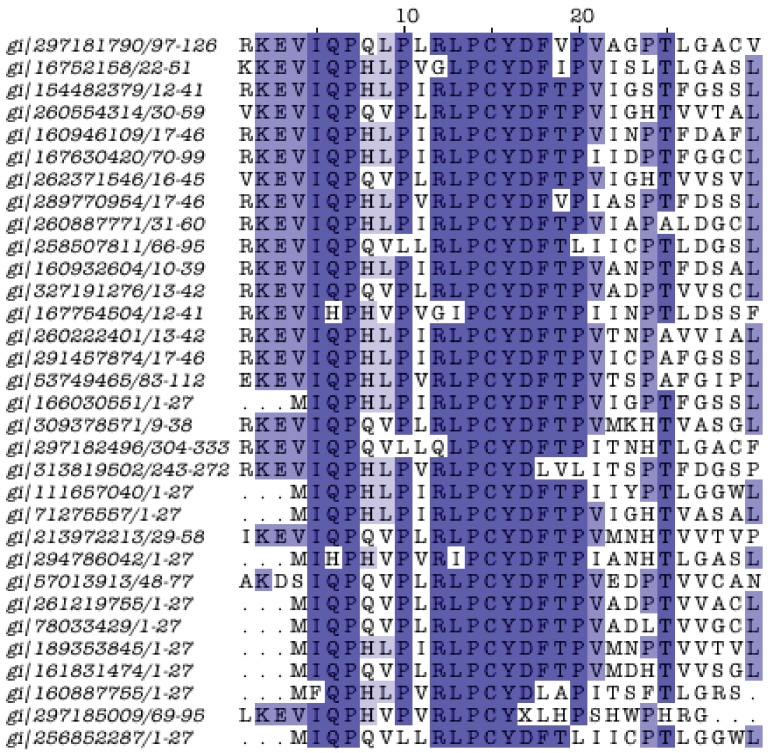
Alignment of the PCY domain. The PCY motif is centered around position 15 of the multiple alignment. The domain was discovered following five iterations with PSI-BLAST with CP0988 as query sequence (GI:16752158), until convergence and an e-value threshold 0.005. In total 70 sequences were recovered; redundancy was removed at 95% with Jalview [34], resulting in 32 sequences shown here. The length of the domain is just 30 residues; boxes signify sequence identity at 50% or above (darker color: more conserved). GI labels are provided, along with sequence coordinates on the left of the alignment (see text for more details and discussion).

One such domain is an enigmatic, short and highly conserved motif containing the triplet Pro-Cys-Tyr (PCY), present in the *C. pneumoniae* AR39 CP0988 protein. This protein is 52 residues long and does not exhibit significant similarities to any other protein in the *Chlamydiales* pangenome. However, it does show similarity to a set of short proteins (<100 residues long) from various species, including *Acinetobacter*, *Brucella*, *Clostridium*, *Coxiella*, *Curvibacter*, *Eubacterium*, *Parvimonas*, *Rhizobium*, *Ruminococcus*, *Selenomonas*, *Streptomyces*, other longer proteins from *Chloroflexi*, *Heliobacterium*, *Lactobacillus*, the *C*-terminus of a *Propionibacterium* protein (HL046PA2) and an uncultured *Acidobacteria* bacterium HF4000_26D02, and importantly, to a number of longer plant proteins from *Nicotiana tabacum*, *Pinus koraiensis*, *Solanum demissum* (middle of protein) and *Vitis vinifera* (*N*-terminus, total length 1,193 residues) (Supplement S8). This conserved region with this peculiar phylogenetic distribution has not been characterized previously to our knowledge, and can be considered a genuine novel domain of unknown function (Figure 4). It remains unclear whether the domain has been universally lost from the *Chlamydiales* pangenome or acquired from *C. pneumoniae* through horizontal transfer.

**Figure 5:**
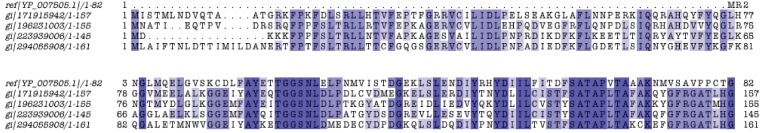
Alignment of a unique leucyl aminopeptidase family. The domain was discovered following five iterations with PSI-BLAST with pc0506 as query sequence (YP_007505.1). Display conventions as in Figure 4.

Another interesting example of a unique protein is the *P. amoebophila* pc0506. This 82-residue-long uncharacterized protein is evidently absent from the core pangenome and yet it exhibits significant similarity to four Verrucomicrobia proteins from *Verrucomicrobium spinosum*, *Chthoniobacter flavus*, *Pedosphaera parvula* and *Coraliomargarita akajimensis*, in this order of similarity, ranging from 53% down to 44% sequence identity (Figure 5). The above mentioned proteins reportedly belong the leucyl aminopeptidase superfamily (Supplement S9). The functional significance of this biochemical role for *P. amoebophila* is not yet understood. Yet, the strong mutual similarity of this protein family with Verrucomicrobial and *P. amoebophila* members (no other member in the entire pangenome) can be placed within the general controversy of the connection of *Chlamydiales* with the so-called PVC group [41,42] (see below).

In all, it appears that properties encoded from most unique genes, apart from their unusual phylogenetic distribution, represent accessory functional roles that provide additional versatility to the largest genomes in the group, possibly related to their extra functional capabilities. Two exceptions with seemingly central functions are wcw_0805, with similarity to the 50S L34 ribosomal protein family and wcw_861, with similarity to 6-pyruvoyl tetrahydrobiopterin synthases, both from *W. chondrophila* WSU (not shown).

### 3.8. Protein Family Contributions from Genome Projects

**Figure 6:**
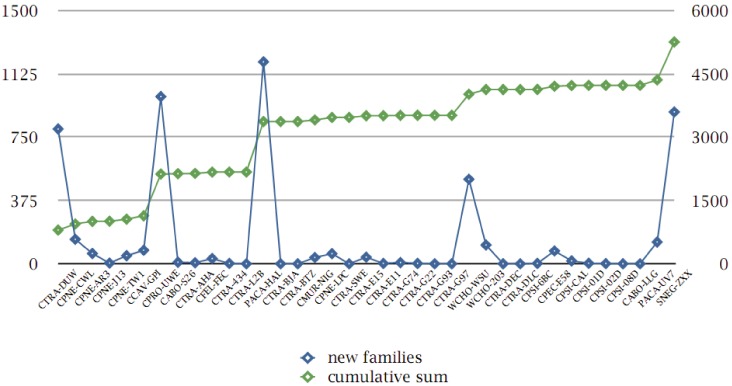
Protein family contributions from genome projects. Genome codes are sorted according to their original publication date (and/or release date, x-axis); absolute number of “novel” protein families within the pangenome are given (left y-axis, blue curve and square symbols); cumulative sum of protein families (up to 5,260, excluding those without self-hits, see text) is also shown, defined as a “pangenome saturation curve” (right y-axis, green curve and square symbols).

As mentioned above, we have tracked the original publication (and/or release) data for the genomes under consideration, in terms of novel families detected per genome sequence (Supplement S10). By mapping the protein families which appear first in this ranking order, we can thus estimate the relative “novelty” or contribution of previously unseen protein families within the chlamydial pangenome and the typical “pangenome saturation curve” (Figure 6).

As expected, and discussed above (Figure 3), for the densest part of the group, little or no contributions have been provided. Apart from the larger genomes, which have added hundreds of new gene types [19], the more distant members of the group with small genomes, for instance *C. caviae* or *C. pecorum*, have also contributed a significant number (80 and 76, respectively—Supplement S10).

### 3.9. Genome Phylogeny

**Figure 7:**
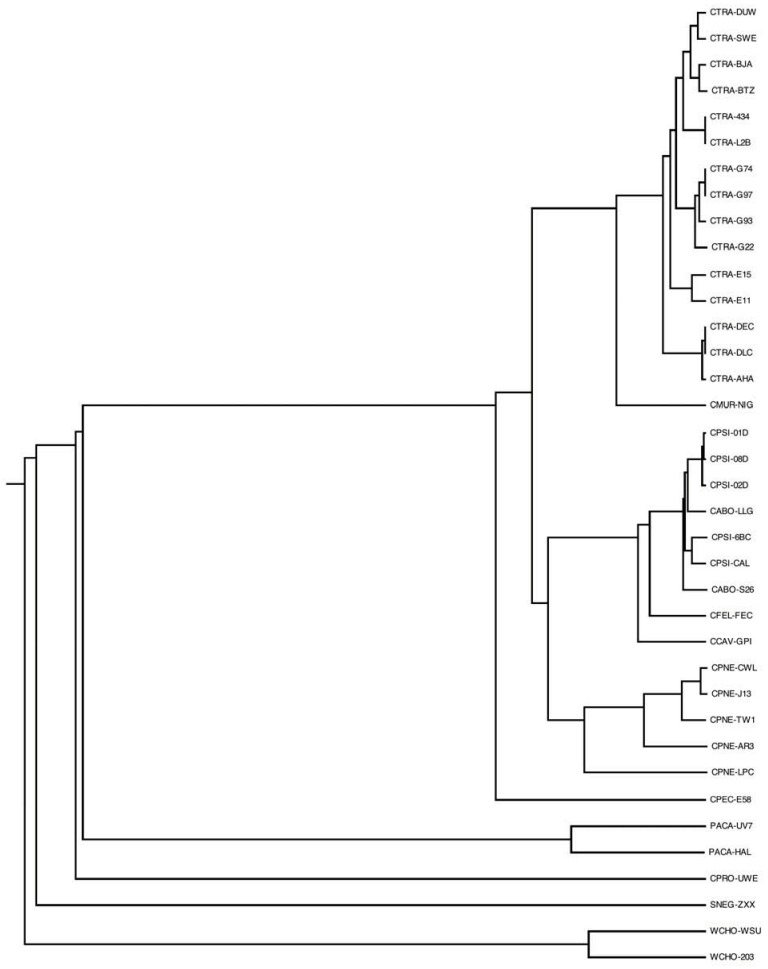
Genome tree of the *Chlamydiales*. Dendrogram representing phylogenetic relationships of the 37 *Chlamydiales* genomes analyzed, based on sharing of phylogenetic profiles (see Experimental Section for details). Genome codes are given as labels.

Finally, we have reconstructed the genome phylogeny of the pangenome based on the sharing of phylogenetic profile patterns based on the above analysis (see Experimental Section). Evidently, the pangenome is stratified according to the known, established phylogeny patterns [10] (Figure 7). The genome tree is another concise way to visualize the “novelty” components of the various species and strains that have been sequenced, exemplified above in various contexts, e.g., number of unique genes (Figure 3) or the tracking of the relative contributions of novel protein families (Figure 6). A future aspect of this work will be to infer the history of the pangenome using methods of ancestral state reconstruction [43]. The evolutionary history of the *Chlamydiales* as reflected by the genome tree might also shed light on the ongoing controversy about their status within the tree of life [41].

The genome tree accurately reflects the current taxonomy of *Chlamydiales* [4,44], with a couple of notable exceptions namely the clustering of *C. abortus* with *C. psittaci*, the closer relationship of *C. felis* with the former two species against *C. caviae*—in agreement with previous findings [6,44] but not with other proposals [8]—as well as the distinct relationship of *C. pecorum* at the root of *Chlamydiacae* and not as a sister group of *C. pneumoniae* [4,44]. The resulting phylogenetic tree using genome-wide phylogenetic profile sharing patterns can also act as an internal control of the pangenome analysis, since all the closely related strains sequenced are grouped together with very high accuracy (Figure 7).

## 4. Discussion

Our results suggest that the *Chlamydiales* pangenome reflects a certain degree of structural stability, as core genes represent over a quarter of an average genome, as well as functional coherence, in the sense that most functional properties of these genes are consistent with current knowledge. Unlike various claims in the recent literature, it turns out that, at least in the case of a highly constrained pangenome of intracellular pathogens, there is an unexpected degree of stability, given the wide range of phylogenetic relationships within this particular taxon.

It is thus shown that for the smallest of genomes (<900 protein-coding genes), over a third of their gene content is shared with larger genomes (>2,000 genes), decorated by a broader element of so-called “character”, or peripheral, genes. This distribution, which in turn is influenced by the sampling of phylogeny and other factors, requires further investigation, being beyond the scope of this work.

It should also be pointed out that the *Chlamydiales* pangenome exhibits general characteristics of distribution not dissimilar to other recent pangenome analyses, including those of the *Salmonella* pangenome with 45 strains [15], the *Streptococcus pneumoniae* pangenome with 44 strains [14] and the *Campylobacter* pangenome with 96 strains [28], suggesting the conservation of a core pangenome within and across bacterial taxa that have been sampled adequately. In the case of *Salmonella*, tracking the contributions of new strains to the entire core set and the pangenome suggests a slight expansion with more sampling and a stable core, reminiscent of the *Chlamydiales*, with one third of the pangenome represented in the core set [15]. A slightly less stable pattern is detected in the *Streptococcus pneumoniae* group [14], possibly due to a wider diversity in that sample, yet with a similar pattern of core set saturation. Interestingly, an attempt for ancestral reconstruction in the *S. pneumoniae*/*S. mitis* complex suggests that there is a dual process of genome expansion and reduction in the different paths leading to the genomes of contemporary strains [14]. A more comprehensive analysis of the *Campylobacter* pangenome with 96 strains [28], using a combination of experimental and theoretical work, also points to the same direction: Within the two species groups examined, the core gene set overlap reaches 80%, supporting earlier findings for the related *Helicobacter pylori* strains [45].

## 5. Conclusions

We have thus examined the salient features of the *Chlamydiales* pangenome, introducing a pangenome analysis pipeline and certain definitions that facilitate the discovery of core and peripheral genes, the identification of unique genes with various origins as well as the detection of novel protein sequence domains. We expect that analogous efforts will lead to rigorous standards for pangenome analysis in the future. Future research opportunities abound, for example: ancestral reconstruction [43], syntenic patterns of genome structure (e.g., [28,45]), the (presently limited) enrichment with expression data, the evolutionary histories of ‘peripheral’ genes (as discussed above), the connection of *Chlamydiales* with plants [46,47,48,49,50], the position of the *Chlamydiales* in the tree of life, and the connection with the PVC superphylum [41,42,50]. Wider challenges that go beyond the above pangenome-specific issues might include a more detailed annotation of the entire dynamic range of family distribution [21], the characterization of protein function in a wider context including comparative metabolic reconstructions [19], the evolution of mobile elements [51], the deeper understanding of the physiological and pathological properties [52,53] of the strains that have been sequenced and the connection with other pangenomes [28].

## Appendix

**Appendix-Table 1:** Core gene and protein families in the *Chlamydiales*.

Cluster ID	Lead Sequence	Master Sequence	Function Annotation from Master Sequence
181	CABO-LLG-01-000000	CTRA-DUW-01-000647	NA
182	CABO-LLG-01-000003	CTRA-DUW-01-000644	ENZYME UDP-*N*-acetylglucosamine pyrophosphorylase [EC] 2.7.7.23
183	CABO-LLG-01-000004	CTRA-DLC-01-000248	FUNCTION PhoB-like protein
184	CABO-LLG-01-000015	CTRA-DLC-01-000228	FUNCTION RecA protein
185	PACA-UV7-01-001616	CTRA-DUW-01-000487	NA
186	CABO-LLG-01-000023	CTRA-DUW-01-000658	NA
187	CABO-LLG-01-000024	CTRA-DUW-01-000624	FUNCTION RNA Polymerase Sigma-54 factor RpoN
188	CABO-LLG-01-000026	CTRA-DUW-01-000622	ENZYME Uracil DNA glycosylase [EC] 3.2.2.-
189	CABO-LLG-01-000028	CTRA-DUW-01-000620	SIMILAR-TO NTPase HAM1 homolog [EC] 3.6.1.15
190	CABO-LLG-01-000029	CTRA-DLC-01-000273	NA
191	CABO-LLG-01-000032	CTRA-DUW-01-000616	NA
192	CABO-LLG-01-000034	CTRA-DLC-01-000278	FUNCTION Peptidoglycan-associated lipoprotein
193	CABO-LLG-01-000035	CTRA-DLC-01-000279	FUNCTION TolB macromolecule uptake homolog
194	CABO-LLG-01-000037	CTRA-DUW-01-000611	FUNCTION TolR/ExbD macromolecule uptake homolog
195	CABO-LLG-01-000040	CTRA-DLC-01-000284	FUNCTION protein translocase TatD/MttC homolog
196	CABO-LLG-01-000047	CTRA-DUW-01-000600	ENZYME enolase [EC] 4.2.1.11
197	CABO-LLG-01-000048	CTRA-DUW-01-000599	FUNCTION Excinuclease ABC subunit B
198	CABO-LLG-01-000049	CTRA-DUW-01-000598	ENZYME Tryptophanyl-tRNA Synthetase [EC] 6.1.1.2
199	CTRA-G22-01-000161	CTRA-DUW-01-000746	ENZYME Seryl-tRNA Synthetase [EC] 6.1.1.11
200	CABO-LLG-01-000054	CTRA-DUW-01-000593	FUNCTION Nickel transporter CnrT homolog
201	CABO-LLG-01-000061	CTRA-DUW-01-000586	NA
202	CABO-LLG-01-000062	CTRA-DUW-01-000585	FUNCTION type II secretion system protein D homolog
203	CABO-LLG-01-000063	CTRA-DUW-01-000584	FUNCTION type II secretion system protein E homolog
204	CABO-LLG-01-000064	CTRA-DLC-01-000308	FUNCTION type II secretion system protein F homolog
205	CABO-LLG-01-000065	CTRA-DLC-01-000309	NA
206	CABO-LLG-01-000070	CTRA-DUW-01-000577	FUNCTION protein secretion system YscT homolog
207	CABO-LLG-01-000072	CTRA-DLC-01-000316	FUNCTION protein secretion system YscR homolog
208	CABO-LLG-01-000073	CTRA-DEC-01-000317	FUNCTION protein secretion system YscL homolog
209	CABO-LLG-01-000074	CTRA-DUW-01-000573	NA
210	CABO-LLG-01-000076	CTRA-DUW-01-000571	ENZYME lipoate synthase [EC] 2.8.1.-
211	CABO-LLG-01-000081	CTRA-DUW-01-000714	ENZYME Endonuclease III [EC] 4.2.99.18
212	CABO-LLG-01-000083	CTRA-DUW-01-000716	ENZYME Phosphatidylserine decarboxylase [EC] 4.1.1.65
213	CABO-LLG-01-000085	CTRA-DUW-01-000718	FUNCTION preprotein translocase subunit SecA
214	CABO-LLG-01-000089	CTRA-DUW-01-000722	ENZYME ATP-dependent Clp protease ATP-binding subunit ClpX [EC]
215	CABO-LLG-01-000091	CTRA-DUW-01-000724	FUNCTION Trigger factor
216	CABO-LLG-01-000093	CTRA-DUW-01-000726	FUNCTION Rod shape-determining protein MreB
217	CABO-LLG-01-000094	CTRA-DUW-01-000727	ENZYME Phosphoenolpyruvate carboxykinase (GTP) [EC] 4.1.1.32
218	CABO-LLG-01-000098	CTRA-DUW-01-000731	ENZYME Glycerol-3-phosphate dehydrogenase [NAD+] [EC] 1.1.1.8
219	CABO-LLG-01-000099	CTRA-DUW-01-000732	ENZYME UDP-N-acetylhexosamine pyrophosphorylase [EC] 2.7.7.-
220	CCAV-GPI-01-000128	CTRA-DUW-01-000503	FUNCTION Transcription termination factor Rho
221	CABO-LLG-01-000104	CTRA-DUW-01-000737	DOMAIN NifU
222	PACA-HAL-01-002518	CTRA-DUW-01-000261	ENZYME NifS aminotransferase [EC] -.-.-.-
223	CABO-LLG-01-000109	CTRA-DUW-01-000742	ENZYME Biotin-[acetyl-CoA-carboxylase] synthetase [EC] 6.3.4.15
224 *	CABO-LLG-01-000121	CTRA-DUW-01-000754	DOMAIN SET
225	CABO-LLG-01-000122	CTRA-DUW-01-000755	SIMILAR-TO metallo-beta-lactamase [EC] 3.5.-.-
226	CABO-LLG-01-000123	CTRA-DUW-01-000756	FUNCTION Cell division protein FtsK C-terminus
227	CABO-LLG-01-000125	CTRA-DUW-01-000757	NA
228	CABO-LLG-01-000126	CTRA-DUW-01-000758	FUNCTION preprotein translocase complex subunit YajC
229	CABO-LLG-01-000130	CTRA-DUW-01-000762	ENZYME Protoporphyrinogen oxidase HemY [EC] 1.3.3.4
230	CABO-LLG-01-000132	CTRA-DUW-01-000764	ENZYME Uroporphyrinogen decarboxylase HemE [EC] 4.1.1.37
231	CABO-LLG-01-000134	CTRA-DLC-01-000129	ENZYME Alanyl-tRNA Synthetase [EC] 6.1.1.7
232	CABO-LLG-01-000135	CTRA-DUW-01-000767	ENZYME Transketolase [EC] 2.2.1.1
233	CABO-LLG-01-000136	CTRA-DUW-01-000768	SIMILAR-TO AMP nucleosidase [EC] 3.2.2.4
234	CABO-LLG-01-000142	CTRA-DUW-01-000774	ENZYME Phospho-*N*-acetylmuramoyl-pentapeptide-transferase [EC]
235	CABO-LLG-01-000143	CTRA-DUW-01-000775	ENZYME UDP-*N*-acetylmuramoylalanine-D-glutamate ligase [EC]
236	CABO-LLG-01-000144	CTRA-DUW-01-000776	SIMILAR-TO *N*-acetylmuramoyl-L-alanine amidase *C*-terminus [EC]
237	CABO-LLG-01-000146	CTRA-DLC-01-000117	ENZYME UDP-*N*-acetylglucosamine-*N*-acetylmuramyl-(pentapeptide) pyrophosphoryl-undecaprenol *N*-acetylglucosamine transferase
238	CABO-S26-01-000517	CTRA-DUW-01-000125	ENZYME Biotin carboxylase [EC] 6.3.4.14
239	CABO-LLG-01-000150	CTRA-DUW-01-000781	NA
240	CABO-LLG-01-000155	CTRA-DUW-01-000786	NA
241	CABO-LLG-01-000157	CTRA-DUW-01-000788	ENZYME bis(5'-nucleosyl)-tetraphosphatase [EC] 3.6.1.17
242	CABO-LLG-01-000168	CTRA-DLC-01-000098	ENZYME Cysteinyl-tRNA Synthetase [EC] 6.1.1.16
243	CABO-LLG-01-000173	CTRA-DUW-01-000804	FUNCTION Ribosomal protein S14
244	CABO-LLG-01-000174	CTRA-DUW-01-000805	NA
245	CABO-LLG-01-000176	CTRA-DUW-01-000808	ENZYME Excinuclease ABC subunit C [EC] -.-.-.-
246	CABO-LLG-01-000177	CTRA-DUW-01-000809	FUNCTION DNA mismatch repair protein MutS
247	CABO-LLG-01-000184	CTRA-DUW-01-000815	ENZYME CDP-diacylglycerol-glycerol-3-phosphate
248	CABO-LLG-01-000185	CTRA-DUW-01-000816	ENZYME Glycogen synthase [EC] 2.4.1.21 2
249	CABO-LLG-01-000186	CTRA-DUW-01-000817	FUNCTION Ribosomal protein L25
250	CABO-LLG-01-000187	CTRA-DUW-01-000818	ENZYME Peptidyl-tRNA hydrolase [EC] 3.1.1.29
251	CABO-LLG-01-000188	CTRA-DUW-01-000819	FUNCTION Ribosomal protein S6
252	CABO-LLG-01-000189	CTRA-DUW-01-000820	FUNCTION Ribosomal protein S18
253	CABO-LLG-01-000190	CTRA-DUW-01-000821	FUNCTION Ribosomal protein L9
254 *	CABO-LLG-01-000193	CTRA-DUW-01-000823	NA
255 *	CABO-LLG-01-000194	CTRA-DUW-01-000824	SIMILAR-TO Small-peptide endopeptidase [EC] 3.4.24.55
256	CABO-LLG-01-000195	CTRA-DLC-01-000073	ENZYME Glycerol-3-phosphate acyltransferase [EC] 2.3.1.15
257	CABO-LLG-01-000196	CTRA-DLC-01-000072	ENZYME Ribonuclease E [EC] 3.1.4.-
258	CABO-LLG-01-000197	CTRA-DLC-01-000071	NA
259	CABO-LLG-01-000214	CTRA-DLC-01-000063	ENZYME Glucosamine-fructose-6-phosphate aminotransferase [EC]
260	CABO-LLG-01-000218	CTRA-DUW-01-000840	ENZYME Succinyl-CoA synthetase beta chain [EC] 6.2.1.5
261	CABO-LLG-01-000222	CTRA-DUW-01-000843	SIMILAR-TO Small-peptide endopeptidase [EC] 3.4.24.55
262	CABO-LLG-01-000224	CTRA-DUW-01-000845	ENZYME CDP-diacylglycerol-serine *O*-phosphatidyltransferase [EC]
263	CABO-LLG-01-000229	CTRA-DUW-01-000850	ENZYME UDP-*N*-acetylenolpyruvoylglucosamine reductase [EC]
264	CABO-LLG-01-000230	CTRA-DLC-01-000047	FUNCTION Transcription termination protein NusB
265	CABO-LLG-01-000231	CTRA-DLC-01-000046	NA
266	CABO-LLG-01-000233	CTRA-DUW-01-000854	FUNCTION Ribosomal protein L20
267	CABO-LLG-01-000234	CTRA-DUW-01-000855	ENZYME Phenylalanyl-tRNA Synthetase alpha chain [EC] 6.1.1.20
268	CABO-LLG-01-000236	CTRA-DUW-01-000857	NA
269	CABO-LLG-01-000237	CTRA-DUW-01-000858	NA
270	CABO-LLG-01-000240	CTRA-DUW-01-000861	ENZYME Polynucleotide phosphorylase [EC] 2.7.7.8
271	CABO-LLG-01-000241	CTRA-DUW-01-000862	NA
272 *	CABO-LLG-01-000254	CTRA-DUW-01-000874	FUNCTION ABC transporter, ATP-binding protein *N*-terminus
273	CABO-LLG-01-000267	CTRA-DUW-01-000385	ENZYME Glucose-6-phosphate isomerase [EC] 5.3.1.9
274	CABO-LLG-01-000269	CTRA-DLC-01-000502	ENZYME Malate dehydrogenase [EC] 1.1.1.82
275	CABO-LLG-01-000271	CTRA-DUW-01-000382	SIMILAR-TO D-Amino Acid Dehydrogenase [EC] 1.-.-.-
276 *	CABO-LLG-01-000276	CTRA-DLC-01-000508	ENZYME 3-dehydroquinate dehydratase [EC] 4.2.1.10
277	CPRO-UWE-01-000881	CTRA-DUW-01-000373	ENZYME 3-phosphoshikimate 1-carboxyvinyltransferase [EC]
278	CABO-LLG-01-000277	CTRA-DUW-01-000376	ENZYME 3-dehydroquinate synthase [EC] 4.6.1.3
279	CABO-LLG-01-000278	CTRA-DUW-01-000375	ENZYME Chorismate synthase [EC] 4.6.1.4
280	CABO-LLG-01-000288	CTRA-DUW-01-000371	ENZYME Dihydrodipicolinate reductase [EC] 1.3.1.26
281	CABO-LLG-01-000290	CTRA-DUW-01-000369	ENZYME Aspartokinase [EC] 2.7.2.4
282	CABO-LLG-01-000298	CTRA-DUW-01-000328	NA
283	SNEG-ZXX-01-000625	CTRA-DLC-01-000783	FUNCTION Translation initiation factor IF-2
284	CABO-LLG-01-000304	CTRA-DUW-01-000322	FUNCTION Ribosomal protein L11
285	CABO-LLG-01-000305	CTRA-DUW-01-000321	FUNCTION Ribosomal protein L1
286	CABO-LLG-01-000306	CTRA-DUW-01-000320	FUNCTION Ribosomal protein L10
287	CABO-LLG-01-000308	CTRA-DUW-01-000318	ENZYME DNA-directed RNA polymerase beta subunit [EC] 2.7.7.6
288	CABO-LLG-01-000309	CTRA-DUW-01-000317	ENZYME DNA-directed RNA polymerase beta prime subunit [EC]
289	CABO-LLG-01-000312	CTRA-DUW-01-000314	NA
290	CABO-LLG-01-000313	CTRA-DLC-01-000569	ENZYME vacuolar ATPase proteolipid subunit E [EC] 3.6.1.34
291	CABO-LLG-01-000314	CTRA-DUW-01-000312	NA
292	CABO-LLG-01-000317	CTRA-DUW-01-000309	ENZYME vacuolar ATPase proteolipid subunit D [EC] 3.6.1.34
293	CABO-LLG-01-000320	CTRA-DLC-01-000576	NA
294	CABO-LLG-01-000324	CTRA-DUW-01-000337	ENZYME Pyruvate kinase [EC] 2.7.1.40
295	CPRO-UWE-01-001632	CTRA-DUW-01-000012	NA
296	CABO-LLG-01-000328	CTRA-DUW-01-000013	ENZYME Cytochrome Oxidase D subunit I [EC] 1.10.3.-
297	CABO-LLG-01-000329	CTRA-DUW-01-000014	ENZYME Cytochrome Oxidase D subunit II [EC] 1.10.3.-
298	CABO-LLG-01-000331	CTRA-DLC-01-000860	NA
299	CABO-LLG-01-000332	CTRA-DLC-01-000861	NA
300	CABO-LLG-01-000333	CTRA-DUW-01-000015	FUNCTION PhoH-like protein
301	CABO-LLG-01-000337	CTRA-DLC-01-000856	NA
302	CABO-LLG-01-000338	CTRA-DUW-01-000022	FUNCTION Ribosomal protein L31
303	CABO-LLG-01-000342	CTRA-DUW-01-000026	FUNCTION Ribosomal protein S16
304	CABO-LLG-01-000343	CTRA-DUW-01-000027	ENZYME tRNA (guanine *N*-1) methyltransferase [EC] 2.1.1.31
305	CABO-LLG-01-000344	CTRA-DUW-01-000028	FUNCTION Ribosomal protein L19
306	CABO-LLG-01-000345	CTRA-DUW-01-000029	ENZYME Ribonuclease HII [EC] 3.1.26.4
307	CABO-LLG-01-000346	CTRA-DUW-01-000030	ENZYME Guanylate kinase [EC] 2.7.4.8
308	CABO-LLG-01-000358	CTRA-DUW-01-000215	ENZYME Ribose 5-phosphate isomerase A [EC] 5.3.1.6
309	CABO-LLG-01-000359	CTRA-DLC-01-000666	NA
310	CABO-LLG-01-000360	CTRA-DUW-01-000213	NA
311	CABO-LLG-01-000368	CTRA-DLC-01-000765	NA
312	CABO-LLG-01-000374	CTRA-DUW-01-000147	ENZYME DNA ligase (NAD+) [EC] 6.5.1.2
313	CABO-LLG-01-000379	CTRA-DUW-01-000210	ENZYME 3-deoxy-d-manno-octulosonic-acid transferase [EC] 2.-.-.-
314	CABO-LLG-01-000392	CTRA-DUW-01-000186	NA
315	CABO-LLG-01-000393	CTRA-DUW-01-000185	ENZYME CTP synthetase [EC] 6.3.4.2
316	CABO-LLG-01-000404	CTRA-DUW-01-000195	ENZYME Queuine tRNA-ribosyltransferase [EC] 2.4.2.29
317	CABO-LLG-01-000420	CTRA-DUW-01-000132	NA
318	CABO-LLG-01-000423	CTRA-DUW-01-000199	ENZYME *O*-sialoglycoprotein endopeptidase [EC] 3.4.24.57
319	CABO-LLG-01-000425	CTRA-DUW-01-000187	NA
320	CABO-LLG-01-000426	CTRA-DUW-01-000188	ENZYME Glucose-6-phosphate dehydrogenase [EC] -.-.-.-
321	CABO-LLG-01-000432	CTRA-DLC-01-000753	FUNCTION Ribosomal protein S9
322	CABO-LLG-01-000433	CTRA-DUW-01-000126	FUNCTION Ribosomal protein L13
323	CABO-LLG-01-000435	CTRA-DUW-01-000152	NA
324	CABO-LLG-01-000448	CTRA-DUW-01-000138	FUNCTION Sua5 homolog
325	CABO-LLG-01-000451	CTRA-DUW-01-000190	ENZYME Thymidylate kinase (dTMP kinase) [EC] 2.7.4.9
326	CABO-LLG-01-000459	CTRA-DUW-01-000217	ENZYME Fructose-bisphosphate aldolase class I [EC] 4.1.2.13
327	CABO-LLG-01-000471	CTRA-DUW-01-000239	FUNCTION acyl carrier protein ACP
328	PACA-UV7-01-000731	CTRA-DUW-01-000105	ENZYME Enoyl-[acyl-carrier protein] reductase (NADH) [EC]
329	CABO-LLG-01-000473	CTRA-DLC-01-000640	ENZYME Malonyl CoA-acyl carrier protein transacylase [EC]
330	CABO-LLG-01-000474	CTRA-DLC-01-000639	ENZYME 3-oxoacyl-[acyl-carrier-protein] synthase III [EC]
331	CABO-LLG-01-000475	CTRA-DUW-01-000243	FUNCTION Recombination protein RecR homolog
332	CABO-LLG-01-000477	CTRA-DUW-01-000245	NA
333	CPNE-TW1-01-000387	CTRA-DUW-01-000055	ENZYME 2-oxoglutarate dehydrogenase E1 component [EC] 1.2.4.2
334	CABO-LLG-01-000486	CTRA-DUW-01-000254	FUNCTION Inner-membrane protein YidC
335	CABO-LLG-01-000489	CTRA-DUW-01-000101	ENZYME holo-[acyl-carrier protein] synthase [EC] 2.7.8.7
336	CABO-LLG-01-000490	CTRA-DUW-01-000100	ENZYME Thioredoxin reductase (NADPH) [EC] 1.6.4.5
337	CABO-LLG-01-000494	CTRA-DUW-01-000096	FUNCTION Ribosome-binding factor A RbfA
338	CABO-LLG-01-000496	CTRA-DUW-01-000094	ENZYME Riboflavin kinase [EC] 2.7.1.26
339	CABO-LLG-01-000499	CTRA-DUW-01-000090	NA
340	CABO-LLG-01-000500	CTRA-DUW-01-000089	NA
341	CABO-LLG-01-000501	CTRA-DLC-01-000793	FUNCTION Ribosomal protein L28
342	CABO-LLG-01-000508	CTRA-DLC-01-000801	ENZYME Methylenetetrahydrofolate dehydrogenase [EC] 1.5.1.15
343	CABO-LLG-01-000509	CTRA-DUW-01-000078	FUNCTION Thiamine biosynthesis lipoprotein ApbE precursor
344	CABO-LLG-01-000510	CTRA-DUW-01-000077	FUNCTION Small protein B SmpB homolog
345	CABO-LLG-01-000511	CTRA-DUW-01-000076	ENZYME DNA polymerase III beta chain [EC] 2.7.7.7
346	CABO-LLG-01-000514	CTRA-DUW-01-000073	SIMILAR-TO zinc protease [EC] -.-.-.-
347	CABO-LLG-01-000516	CTRA-DUW-01-000071	FUNCTION ABC transporter, permease protein TroD
348	CABO-LLG-01-000519	CTRA-DUW-01-000068	FUNCTION periplasmic substrate binding protein TroA
349	CPSI-CAL-01-000799	CTRA-DUW-01-000423	FUNCTION high-affinity ZnuA homolog
350	CABO-LLG-01-000520	CTRA-DLC-01-000812	NA
351	CABO-LLG-01-000523	CTRA-DUW-01-000064	ENZYME 6-phosphogluconate dehydrogenase [EC] 1.1.1.44
352	CABO-LLG-01-000524	CTRA-DUW-01-000063	ENZYME Tyrosyl-tRNA Synthetase [EC] 6.1.1.1
353	CABO-LLG-01-000535	CTRA-DLC-01-000825	NA
354	CABO-LLG-01-000541	CTRA-DLC-01-000831	NA
355	CABO-LLG-01-000544	CTRA-DUW-01-000045	FUNCTION single-stranded DNA-binding protein SSB
356	CABO-LLG-01-000545	CTRA-DUW-01-000044	NA
357	CABO-LLG-01-000547	CTRA-DUW-01-000042	NA
358	CABO-LLG-01-000554	CTRA-DLC-01-000619	ENZYME Protein phosphatase 2C [EC] 3.1.3.16
359	CABO-LLG-01-000558	CTRA-DUW-01-000257	NA
360	CABO-LLG-01-000560	CTRA-DUW-01-000108	ENZYME A/G-specific adenine glycosylase [EC] 3.2.2.-
361	CABO-LLG-01-000564	CTRA-DUW-01-000104	NA
362	CABO-LLG-01-000571	CTRA-DUW-01-000268	ENZYME Acetyl-coenzyme A carboxylase carboxyl transferase
363	CABO-LLG-01-000574	CTRA-DUW-01-000271	ENZYME N-acetylmuramoyl-L-alanine amidase AmiB [EC] 3.5.1.28
364	CABO-LLG-01-000577	CTRA-DUW-01-000273	FUNCTION Penicillin-binding protein 3
365	CABO-LLG-01-000578	CTRA-DUW-01-000274	NA
366	CABO-LLG-01-000581	CTRA-DUW-01-000277	DOMAIN TPR
367	CABO-LLG-01-000585	CTRA-DLC-01-000601	NA
368	CABO-LLG-01-000586	CTRA-DUW-01-000282	NA
369	CABO-LLG-01-000587	CTRA-DLC-01-000599	NA
370	CPEC-E58-01-000614	CTRA-DUW-01-000284	NA
371	CABO-LLG-01-000591	CTRA-DLC-01-000597	FUNCTION Glycine cleavage system H protein
372	CABO-LLG-01-000594	CTRA-DUW-01-000288	SIMILAR-TO Lipoate-protein ligase A [EC] 6.3.4.-
373	CABO-LLG-01-000596	CTRA-DUW-01-000290	ENZYME tRNA (5-methylaminomethyl-2-thiouridylate)-methyltransferase
374	CABO-LLG-01-000601	CTRA-DUW-01-000293	ENZYME Nitrogen regulatory IIA protein A component [EC] 2.7.1.69
375	WCHO-WSU-01-000243	CTRA-DUW-01-000294	ENZYME Nitrogen regulatory IIA protein A component [EC] 2.7.1.69
376	CABO-LLG-01-000603	CTRA-DUW-01-000295	ENZYME dUTP pyrophosphatase [EC] 3.6.1.23
377	CABO-LLG-01-000608	CTRA-DUW-01-000300	ENZYME Ribonuclease III [EC] 3.1.26.3
378	CABO-LLG-01-000609	CTRA-DLC-01-000581	FUNCTION DNA repair protein RadA
379	CABO-LLG-01-000610	CTRA-DUW-01-000302	ENZYME Porphobilinogen deaminase [EC] 4.3.1.8
380	CABO-LLG-01-000616	CTRA-DUW-01-000340	NA
381	CABO-LLG-01-000623	CTRA-DUW-01-000346	DOMAIN DnaJ
382	CABO-LLG-01-000624	CTRA-DLC-01-000536	FUNCTION Ribosomal protein S21
383	CABO-LLG-01-000628	CTRA-DUW-01-000351	ENZYME Aryl-sulfate sulphohydrolase [EC] 3.1.6.1
384	CABO-LLG-01-000631	CTRA-DUW-01-000354	FUNCTION Septum formation protein Maf homolog
385	CABO-LLG-01-000632	CTRA-DUW-01-000355	NA
386	WCHO-WSU-01-000567	CTRA-DUW-01-000392	NA
387	CABO-LLG-01-000633	CTRA-DUW-01-000356	NA
388	CABO-LLG-01-000636	CTRA-DUW-01-000333	ENZYME Triosephosphate isomerase [EC] 5.3.1.1
389	CABO-LLG-01-000637	CTRA-DUW-01-000334	ENZYME Exonuclease VII large subunit [EC] 3.1.11.6
390	CABO-LLG-01-000641	CTRA-DUW-01-000360	ENZYME Dimethyladenosine transferase [EC] 2.1.1.-
391	CABO-LLG-01-000642	CTRA-DUW-01-000361	NA
392	CABO-LLG-01-000643	CTRA-DUW-01-000362	DOMAIN Thioredoxin
393	CABO-LLG-01-000646	CTRA-DLC-01-000868	NA
394	CABO-LLG-01-000647	CTRA-DLC-01-000869	ENZYME Ribonuclease HII [EC] 3.1.26.4
395	CABO-LLG-01-000651	CTRA-DUW-01-000004	ENZYME glutamyl-tRNA (Gln) amidotransferase, subunit B [EC]
396	CABO-LLG-01-000670	CTRA-DUW-01-000386	NA
397	CABO-LLG-01-000671	CTRA-DUW-01-000387	SIMILAR-TO metallo-beta-lactamase [EC] 3.5.-.-
398	CABO-LLG-01-000684	CTRA-DLC-01-000488	NA
399	CABO-LLG-01-000686	CTRA-DUW-01-000396	NA
400	CABO-LLG-01-000690	CTRA-DLC-01-000484	FUNCTION Heat-inducible transcription repressor HrcA
401	CABO-LLG-01-000691	CTRA-DUW-01-000403	FUNCTION GrpE protein
402	CABO-LLG-01-000698	CTRA-DUW-01-000432	NA
403	CABO-LLG-01-000701	CTRA-DUW-01-000435	NA
404	CABO-LLG-01-000705	CTRA-DUW-01-000438	ENZYME ubiquinone/menaquinone biosynthesis methlytransferase
405	CABO-LLG-01-000706	CTRA-DLC-01-000449	NA
406	CABO-LLG-01-000707	CTRA-DUW-01-000440	ENZYME Diaminopimelate epimerase [EC] 5.1.1.7
407	CABO-LLG-01-000709	CTRA-DLC-01-000446	ENZYME Serine hydroxymethyltransferase [EC] 2.1.2.1
408	CABO-LLG-01-000713	CTRA-DUW-01-000406	NA
409	CABO-LLG-01-000714	CTRA-DLC-01-000479	NA
410	CABO-LLG-01-000717	CTRA-DUW-01-000410	ENZYME Lipid A 4'-kinase [EC] 2.7.1.130
411	CABO-LLG-01-000722	CTRA-DLC-01-000471	FUNCTION DnaK suppressor protein
412	CABO-LLG-01-000723	CTRA-DUW-01-000416	ENZYME Lipoprotein signal peptidase [EC] 3.4.23.36
413	CABO-LLG-01-000735	CTRA-DUW-01-000427	FUNCTION Ribosomal protein L27
414	CABO-LLG-01-000736	CTRA-DLC-01-000459	FUNCTION Ribosomal protein L21
415	CABO-LLG-01-000738	CTRA-DUW-01-000444	NA
416	CABO-LLG-01-000739	CTRA-DUW-01-000445	ENZYME Sulfite reductase (NADPH) flavoprotein alpha-component
417	CABO-LLG-01-000740	CTRA-DLC-01-000442	FUNCTION Ribosomal protein S10
418	CABO-LLG-01-000751	CTRA-DUW-01-000456	ENZYME Glutamyl-tRNA Synthetase [EC] 6.1.1.17
419	CABO-LLG-01-000752	CTRA-DLC-01-000431	NA
420 *	CABO-LLG-01-000753		NA
421	CABO-LLG-01-000754	CTRA-DUW-01-000458	ENZYME Single-stranded-DNA-specific exonuclease RecJ [EC]
422	CABO-LLG-01-000759	CTRA-DUW-01-000463	ENZYME Cytidylate kinase [EC] 2.7.4.14
423	CABO-LLG-01-000761	CTRA-DUW-01-000465	ENZYME Arginyl-tRNA Synthetase [EC] 6.1.1.19
424	CABO-LLG-01-000762	CTRA-DUW-01-000466	ENZYME UDP-*N*-acetylglucosamine 1-carboxyvinyltransferase [EC]
425	CABO-LLG-01-000764	CTRA-DUW-01-000468	NA
426	CABO-LLG-01-000778	CTRA-DUW-01-000480	NA
427	CABO-LLG-01-000779	CTRA-DUW-01-000481	NA
428	CABO-LLG-01-000784	CTRA-DUW-01-000486	ENZYME Phenylalanyl-tRNA Synthetase beta chain [EC] 6.1.1.20
429 *	CABO-LLG-01-000789	CTRA-DUW-01-000491	FUNCTION Dipeptide binding protein DppA
430	CABO-LLG-01-000792	CTRA-DUW-01-000496	NA
431	CABO-LLG-01-000793	CTRA-DUW-01-000497	ENZYME Protoheme ferro-lyase [EC] 4.99.1.1
432	CABO-LLG-01-000794	CTRA-DUW-01-000498	FUNCTION Aminoacid-binding periplasmic protein precursor
433	CABO-LLG-01-000795	CTRA-DUW-01-000499	ENZYME HemK modification methylase homolog [EC] -.-.-.-
434	CABO-LLG-01-000796	CTRA-DUW-01-000500	NA
435	CABO-LLG-01-000801	CTRA-DLC-01-000386	DOMAIN ATP-binding
436	CABO-LLG-01-000802	CTRA-DUW-01-000505	ENZYME DNA polymerase I [EC] 2.7.7.7
437	CABO-LLG-01-000803	CTRA-DLC-01-000384	NA
438	CABO-LLG-01-000805	CTRA-DUW-01-000508	ENZYME CDP-diacylglycerol-glycerol-3-phosphate
439	CABO-LLG-01-000807	CTRA-DUW-01-000511	FUNCTION Glucose inhibited division protein A GidA
440	CABO-LLG-01-000808	CTRA-DUW-01-000512	ENZYME Lipoate-protein ligase A [EC] 6.3.4.-
441	CABO-LLG-01-000810	CTRA-DUW-01-000514	ENZYME Holliday Junction DNA Helicase RuvA [EC] -.-.-.-
442	CABO-LLG-01-000811	CTRA-DUW-01-000515	ENZYME Holliday Junction DNA Helicase RuvC [EC] 3.1.22.4
443	CABO-LLG-01-000813	CTRA-DUW-01-000517	NA
444	CABO-LLG-01-000814	CTRA-DUW-01-000518	ENZYME Glyceraldehyde 3-phosphate dehydrogenase [EC] 1.2.1.12
445	CABO-LLG-01-000820	CTRA-DUW-01-000524	FUNCTION Ribosomal protein L15
446	CABO-LLG-01-000821	CTRA-DUW-01-000525	FUNCTION Ribosomal protein S5
447	CABO-LLG-01-000822	CTRA-DUW-01-000526	FUNCTION Ribosomal protein L18
448	CABO-LLG-01-000824	CTRA-DUW-01-000528	FUNCTION Ribosomal protein S8
449	CABO-LLG-01-000825	CTRA-DUW-01-000529	FUNCTION Ribosomal protein L5
450	CABO-LLG-01-000826	CTRA-DUW-01-000530	FUNCTION Ribosomal protein L24
451	CABO-LLG-01-000827	CTRA-DUW-01-000531	FUNCTION Ribosomal protein L14
452	CABO-LLG-01-000828	CTRA-DUW-01-000532	FUNCTION Ribosomal protein S17
453	CABO-LLG-01-000830	CTRA-DUW-01-000534	FUNCTION Ribosomal protein L16
454	CABO-LLG-01-000831	CTRA-DUW-01-000535	FUNCTION Ribosomal protein S3
455	CABO-LLG-01-000833	CTRA-DUW-01-000537	FUNCTION Ribosomal protein S19
456	CABO-LLG-01-000834	CTRA-DUW-01-000538	FUNCTION Ribosomal protein L2
457	CABO-LLG-01-000835	CTRA-DUW-01-000539	FUNCTION Ribosomal protein L23
458	CABO-LLG-01-000836	CTRA-DLC-01-000351	FUNCTION Ribosomal protein L4
459	CABO-LLG-01-000837	CTRA-DUW-01-000541	FUNCTION Ribosomal protein L3
460 *	CABO-LLG-01-000839	CTRA-DUW-01-000543	ENZYME Methionyl-tRNA formyltransferase [EC] 2.1.2.9
461	CABO-LLG-01-000841	CTRA-DUW-01-000545	ENZYME (3R)-hydroxymyristoyl-[acyl carrier protein] dehydratase
462	CABO-LLG-01-000842	CTRA-DLC-01-000345	ENZYME UDP-3-*O*-[3-hydroxymyristoyl] *N*-acetylglucosamine
463	CABO-LLG-01-000843	CTRA-DUW-01-000547	ENZYME apolipoprotein N-acyltransferase [EC] 2.3.1.
464	CABO-LLG-01-000846	CTRA-DUW-01-000550	DOMAIN ATP-binding
465	CABO-LLG-01-000847	CTRA-DUW-01-000551	NA
466	CABO-LLG-01-000849	CTRA-DUW-01-000553	ENZYME rRNA methyltransferase SpoU homolog [EC] -.-.-.-
467	CABO-LLG-01-000852	CTRA-DUW-01-000556	ENZYME Histidyl-tRNA Synthetase [EC] 6.1.1.21
468	CABO-LLG-01-000855	CTRA-DUW-01-000558	ENZYME DNA polymerase III alpha chain [EC] 2.7.7.7
469	CABO-LLG-01-000856	CTRA-DUW-01-000559	NA
470	CABO-LLG-01-000857	CTRA-DLC-01-000331	NA
471	CABO-LLG-01-000858	CTRA-DUW-01-000561	NA
472	CABO-LLG-01-000860	CTRA-DLC-01-000327	ENZYME D-alanyl-D-alanine carboxypeptidase DacF [EC] 3.4.16.4
473	CABO-LLG-01-000865	CTRA-DUW-01-000710	ENZYME Phosphoglycerate kinase [EC] 2.7.2.3
474	CABO-LLG-01-000867	CTRA-DUW-01-000708	FUNCTION Phosphate transport system protein PhoU
475	CABO-LLG-01-000874	CTRA-DUW-01-000703	FUNCTION ABC transporter, ATP-binding protein
476	SNEG-ZXX-01-002117	CTRA-DUW-01-000701	FUNCTION ABC transporter, ATP-binding protein
477	CABO-LLG-01-000880	CTRA-DUW-01-000697	FUNCTION Ribosomal protein S2
478	CABO-LLG-01-000881	CTRA-DLC-01-000198	FUNCTION Translation elongation factor EF-TS
479	CABO-LLG-01-000882	CTRA-DUW-01-000695	ENZYME Uridylate kinase [EC] 2.7.4.
480	CABO-LLG-01-000892	CTRA-DUW-01-000684	NA
481	CABO-LLG-01-000895	CTRA-DUW-01-000681	DOMAIN FHA
482	CABO-LLG-01-000897	CTRA-DUW-01-000679	ENZYME glutamyl-tRNA reductase [EC] 1.2.1.
483	CABO-LLG-01-000904	CTRA-DUW-01-000672	ENZYME KDO-8-phosphate synthetase [EC] 4.1.2.16
484	CABO-LLG-01-000912	CTRA-DLC-01-000254	NA
485	CABO-LLG-01-000913	CTRA-DUW-01-000640	SIMILAR-TO Endonuclease IV [EC] 3.1.21.2
486	CABO-LLG-01-000914	CTRA-DUW-01-000641	FUNCTION Ribosomal protein S4
487	CABO-LLG-01-000916	CTRA-DUW-01-000657	FUNCTION Multidrug-efflux transporter
488	CABO-LLG-01-000917	CTRA-DLC-01-000238	ENZYME Exodeoxyribonuclease V gamma subunit [EC] 3.1.11.5
489	CABO-LLG-01-000920	CTRA-DUW-01-000652	SIMILAR-TO Amino-acid aminotransferase class I [EC] 2.6.1.
490	CABO-LLG-01-000921	CTRA-DUW-01-000651	FUNCTION Transcription Elongation Factor GreA *C*-terminus
491	CABO-LLG-01-000923	CTRA-DUW-01-000649	NA
492	CABO-LLG-01-000924	CTRA-DLC-01-000245	ENZYME Porphobilinogen synthase [EC] 4.2.1.24

* Eight clusters do not contain one member per genome exactly and are marked; these include cluster 420 which does not contain a master sequence from the *C. trachomatis* original annotation dataset; NA: not available. Lead sequence is first sequence found in cluster; master sequence is sequence where annotation is drawn from (see Experimental Section for details).

## Supplementary Files

Supplementary File 1 Supplementary (ZIP, 57274 KB)
